# Barriers to and Facilitators for Acceptance of Comprehensive Clinical Decision Support System–Driven Care Maps for Patients With Thoracic Trauma: Interview Study Among Health Care Providers and Nurses

**DOI:** 10.2196/29019

**Published:** 2022-03-16

**Authors:** Emma K Jones, Alyssa Banks, Genevieve B Melton, Carolyn M Porta, Christopher J Tignanelli

**Affiliations:** 1 Department of Surgery University of Minnesota Minneapolis, MN United States; 2 University of Minnesota Minneapolis, MN United States; 3 School of Nursing University of Minnesota Minneapolis, MN United States; 4 Department of Surgery North Memorial Health Hospital Robbinsdale, MN United States

**Keywords:** clinical decision support systems, rib fractures, trauma, Unified Theory of Acceptance and Use of Technology, human computer interaction

## Abstract

**Background:**

Comprehensive clinical decision support (CDS) care maps can improve the delivery of care and clinical outcomes. However, they are frequently plagued by usability problems and poor user acceptance.

**Objective:**

This study aims to characterize factors influencing successful design and use of comprehensive CDS care maps and identify themes associated with end-user acceptance of a thoracic trauma CDS care map earlier in the process than has traditionally been done. This was a planned adaptive redesign stage of a User Acceptance and System Adaptation Design development and implementation strategy for a CDS care map. This stage was based on a previously developed prototype CDS care map guided by the Unified Theory of Acceptance and Use of Technology.

**Methods:**

A total of 22 multidisciplinary end users (physicians, advanced practice providers, and nurses) were identified and recruited using snowball sampling. Qualitative interviews were conducted, audio-recorded, and transcribed verbatim. Generation of prespecified codes and the interview guide was informed by the Unified Theory of Acceptance and Use of Technology constructs and investigative team experience. Interviews were blinded and double-coded. Thematic analysis of interview scripts was conducted and yielded descriptive themes about factors influencing the construction and potential use of an acceptable CDS care map.

**Results:**

A total of eight dominant themes were identified: alert fatigue (theme 1), automation (theme 2), redundancy (theme 3), minimalistic design (theme 4), evidence based (theme 5), prevent errors (theme 6), comprehensive across the spectrum of disease (theme 7), and malleability (theme 8). Themes 1 to 4 addressed factors directly affecting end users, and themes 5 to 8 addressed factors affecting patient outcomes. More experienced providers prioritized a system that is *easy to use*. Nurses prioritized a system that incorporated evidence into decision support. Clinicians across specialties, roles, and ages agreed that the amount of extra work generated should be minimal and that the system should help them administer optimal care efficiently.

**Conclusions:**

End user feedback reinforces attention toward factors that improve the acceptance and use of a CDS care map for patients with thoracic trauma. Common themes focused on system complexity, the ability of the system to fit different populations and settings, and optimal care provision. Identifying these factors early in the development and implementation process may facilitate user-centered design and improve adoption.

## Introduction

### Background

When designed well and implemented effectively, clinical decision support systems (CDSSs) have been shown to reduce errors in health care delivery and improve outcomes [1-3]. Clinical care maps provide disease-specific assistance to multidisciplinary clinicians to support evidence-based (EB) practice and organize care processes [4,5]. Trauma is associated with significant morbidity and mortality worldwide and variable adherence to EB practices [6-8]. In the case of rib fractures, adherence to EB practices has been shown to reduce mortality up to 3-fold [4,6]. Although trauma care maps are essential in high-volume trauma centers [2,4], most trauma patients in the United States are treated at smaller-volume community hospitals, which may be less familiar with best practices for the treatment of rib fractures [6]. Scalable clinical decision support (CDS) care maps provide an important bridge in this knowledge-to-practice gap to improve care.

Although CDSSs can be an effective tool to improve adherence to EB practices, many of these systems have posed challenges and fallen short of their full potential [6,9-12]. System challenges can most commonly be attributed to poor user design, poor implementation, or poor institutional integration, resulting in systems with low overall user acceptance [12,13]. Many CDSSs are affected by poor usability, resulting in little demonstrable improvement in adoption, in part because of a lack of integration of end-user feedback [14,15]. Improved implementation and development strategies can overcome the cited barriers that now limit acceptance. For example, the *CDS Five Rights* framework [16,17] was developed to address problems frequently encountered during CDS implementation and usage [14]. This framework ensures that planning teams focus on delivering the right information to the right person, at the right time, in the right format, and through the right channel.

The integration of user-centered design (UCD) in CDS development is another element that can improve acceptability and clinician use behavior [18]. Unfortunately, the widespread adoption of UCD is still not routine, with most UCD focusing on iterative pilot-testing during CDS development or postimplementation evaluation [13,18,19]. There is a critical need for the integration of multidisciplinary, qualitative end-user input before formal CDS electronic health record (EHR) development. Unfortunately, few such qualitative studies at this phase of CDS development exist. A recent review by Khairat et al [12] identified only 11 studies using qualitative methods to evaluate user acceptance of CDS. Only 3 of those studies evaluated acceptance during early phases, such as CDS prototyping. Furthermore, it is critical that such analyses are informed by validated theories surrounding technology intention to use behavior, such as the Technology Acceptance Model [20] or the Unified Theory of Acceptance and Use of Technology (UTAUT) [21]. To overcome these limitations, a study by Khairat et al [12] proposed the User Acceptance and System Adaptation Design (UASAD) model for CDS development, implementation, and evaluation. This model suggests acceptance can be maximized by leveraging end-user feedback (ie, quantitative via survey or qualitative) to understand and integrate user expectations and needs in early CDS development.

Despite the understanding that CDSSs improve adherence to EB practices, which in turn improve outcomes for patients with thoracic trauma, only 3 CDSSs have been published to date in thoracic trauma [2,22,23]. Of these 3 CDSSs [2], 1 CDSS is the published initial result of the implementation of the rib fracture CDS care map prototype referred to in our study and the other 2 focused neither on design nor on the implementation of the CDS.

### Objectives

Aligned with the UASAD model for CDS development, our study uses qualitative interviews to guide the adaptive redesign of a previously created prototype CDS care map for patients with rib fractures before the formal build of the CDSS in the EHR. This is one of the earliest studies to evaluate the UASAD model for CDS prototyping and adaptive redesign. The objective of this study is to identify themes associated with end-user acceptance of a prototype CDSS guided by the UTAUT [21] model for thoracic trauma from qualitative interviews of multidisciplinary trauma end users within a 12-hospital Midwest trauma system.

## Methods

### Prototype CDSS Development

**Figure 1:**
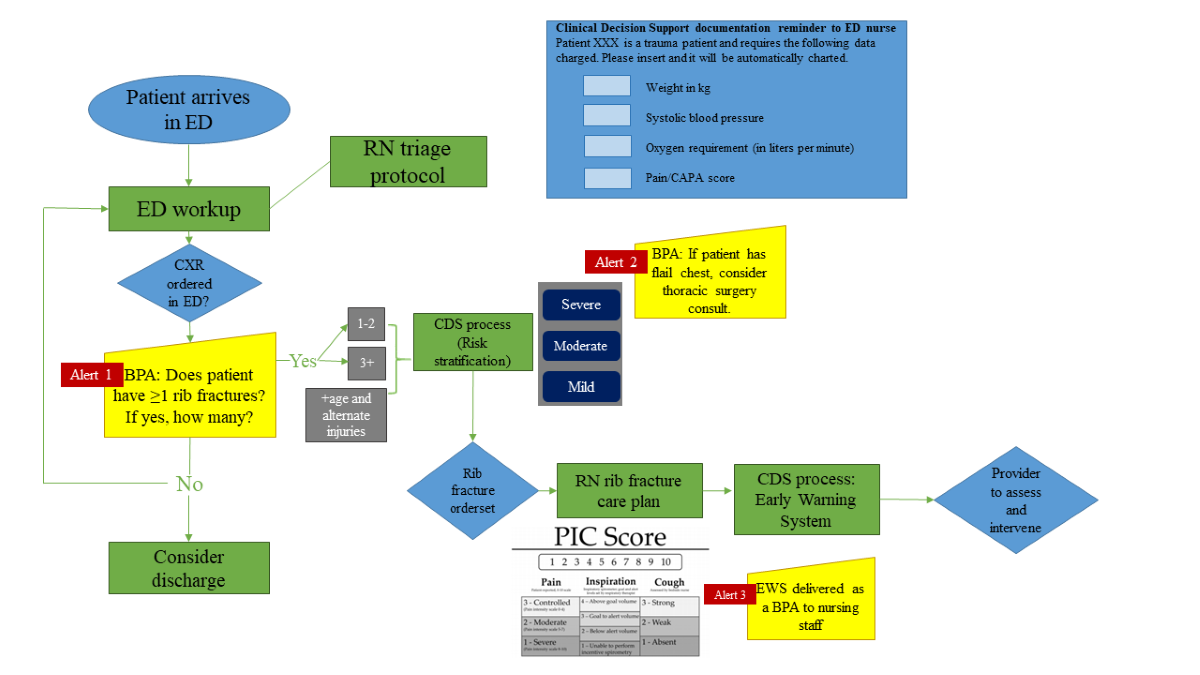
Prototype rib fracture clinical decision support (CDS) care map summary. BPA: best practice advisory; CAPA: clinically aligned pain assessment; CXR: chest x-ray; ED: emergency department; EWS: early warning system; PIC: pain inspiration cough; RN: registered nurse.

A multidisciplinary CDS care map planning team was assembled in April 2018 to address the care of patients with rib fractures. The planning team included 14 members with expertise in trauma surgery, trauma program management and performance improvement, trauma nursing, anesthesiology and pain management, and respiratory therapy. Over a period of 10 months, rib fracture EB practices were cataloged through a formal literature review process that identified 9 peer-reviewed articles and 1 guideline from a regional level 1 trauma center [4,24-32]. A prototype CDS care map was developed, guided by the *CDS Five Rights* framework [17] and validated EB protocols for patients with rib fractures from the American College of Surgeons Trauma Quality Improvement Project, the Eastern Association for the Surgery of Trauma, and the Western Trauma Association [5,26,33-35]. The general workflow of the prototype CDS care map is shown in Figure 1.

### Interview Guide Development and Participants

An interview guide was constructed and included prescripted questions to identify perceptions, behaviors, barriers, and facilitators associated with both general CDS use and our proposed prototype CDSS (Multimedia Appendix 1). Interview guide development was guided by the UTAUT, a validated and widely used model that predicts the behavioral intention to use a technology and is commonly used to assess the likelihood of success for a novel technology [21]. The interview guide was tested with four end users who were not part of the final sample and revised according to their feedback: trauma inpatient registered nurse (RN), trauma advanced practice provider (APP), and 2 trauma medical doctors (MDs). A total of 22 end users comprising trauma MDs, emergency medicine MDs, APPs, and RNs were identified for participation using snowball sampling. In this paper, we use the term *providers* to refer to physicians, nurse practitioners, and physician assistants and we use the term *clinicians* to refer to all providers and nurses.

Participation was voluntary, and informed consent was obtained from participants; all interviews were audio-recorded and stored in a Health Insurance Portability and Accountability Act–compliant research environment. Before participating in the interview, the participants reviewed a 6-minute educational video describing the previously created CDS prototype. All participants completed a brief demographic survey. During the interview, the participants were asked open-ended questions guided by the interview guide. As appropriate, probing questions were asked to examine specific barriers to and facilitators for specific CDS elements.

### Thematic Analysis

Interviews were transcribed verbatim using transcription software (Tybee Types Inc). A coding scheme was generated using prespecified codes based on the UTAUT constructs: performance expectancy, effort expectancy, social influence, and facilitating conditions; emergent codes were added as appropriate. The 2 coders (AB and CJT) met weekly to develop the coding scheme and codebook. Interrater reliability was assessed through a blinded independent coding process between the 2 coders, and coding discrepancies between the coders were resolved through discussion. Following an acceptable level of agreement (>85%), all transcripts were double-coded. All transcripts were coded using computer-assisted qualitative data analysis software (NVivo). A descriptive thematic analysis approach, best described by Hsieh and Shannon [36] as conventional content analysis, was used to categorize the codes into barriers to and facilitating factors for acceptability and assess end users’ intention to use the CDSS and their perspectives on the potential value of this tool.

Although interviews were semistructured, it is important to point out that specific questions regarding each of the themes were not asked; thus, the percentage of clinicians who supported each theme reflects only those clinicians who brought up a topic relating to the theme of their own volition. Therefore, the percentage values reflect the lowest possible number of clinicians, and we cannot say whether we would have had a stronger consensus had clinicians been prompted to comment on topics relating to each theme.

### Ethics Approval

This study was approved by the University of Minnesota institutional review board (STUDY00005353).

## Results

### Participants

A total of 22 trauma clinicians participated, including physicians, APPs, and RNs, who were end users of the EHR. Of the 22 trauma clinicians, 11 (50%) were physicians: 3 (14%) were residents, and 8 (36%) were attending physicians, of whom all but 2 (75%) had been in practice for more than 10 years. Of the other 11 participants, 3 (27%) were APPs, of whom 1 (33%) had been practicing for more than 4 years, and 8 (73%) were RNs, of whom 6 (75%) had ≥10 years of experience. Age of the participants varied from 29 to 62 years, 73% identified as White, 9% as African American, 5% as Latinx, and 5% as multiracial. Interviewees came from a variety of practice models ranging from academic to community practice, and all but one were considered either emergency medicine or trauma surgery primary.

### Thematic Analysis

**Figure 2:**
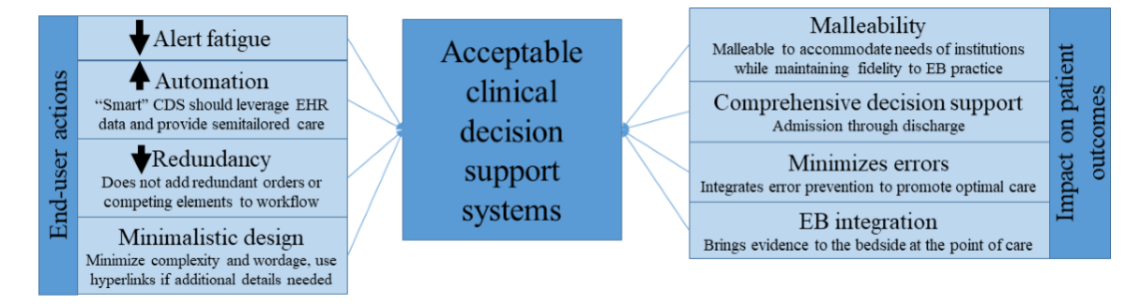
Eight themes for the development of acceptable clinical decision support systems (CDSS). CDS: clinical decision support; EB: evidence based; EHR: electronic health record.

The following eight major themes summarize the overarching concerns and opportunities regarding CDSSs: (1) alert fatigue, (2) automation, (3) reducing redundancy, (4) minimalistic design, (5) EB, (6) promote optimal care and prevent errors, (7) comprehensive across a spectrum of disease or injury, and (8) malleability. Each theme primarily focused on end-user actions or patient outcomes (Figure 2).

Themes summarizing factors directly affecting end users include (1) alert fatigue, (2) automation, (3) reducing redundancy, and (4) minimalistic design. End users desire a *smart* experience, where orders that are already present (eg, activity, nursing, and diet orders) are not duplicated; orders are prepopulated in a risk-stratified and EB manner; and interruptive alerts or *hard stops* are only provided when absolutely necessary. Themes focused on patient outcomes include (5) EB decision support including integration of links to the evidence, practice guidelines, or web-based medical calculators; (6) promote optimal care and prevent errors; (7) comprehensive across a spectrum of disease or injury; and (8) malleability. The final theme encouraged a CDS design that allowed flexibility for each site to customize decision support and recommendations to their institutional capabilities.

We have included 1 to 2 direct quotes from qualitative interviews to illustrate each theme.

#### Theme 1: Alert Fatigue

The most common concern regarding CDSSs is alert fatigue. Of the 22 clinicians, 19 (86%) specifically pointed out alert fatigue as a major problem with other CDS tools they have used in practice. Their concerns surround the frequency of alerts firing, the firing of alerts at inappropriate times, and the rigidity of some alerts:

The other question I’ve got for you is: are you going to have a pop-up for rib fractures, another pop-up for spleen injury, another pop-up for femur fracture, where do you stop with the pop-ups? I’m not going to want to click through 25 pop-ups to write my admission orders.

MD, trauma, >10 years in practice

The hard-stop pop-ups are the most frustrating pop-ups because the computer does not have any judgment, so if you put a hard stop there, that computer is stopped. You can’t order any more orders on your patient until you clear the pop-up. Sometimes you end up ordering something that’s not appropriate for the patient just so you can continue with writing your orders.

MD, trauma, >10 years in practice

More experienced trauma physicians (attending physicians, 4/8, 50%) are skeptical about the added workload that CDS alerts cause and worry that they would have to find ways to work outside the system to maintain efficiency. Of the 14 providers, 6 (43%) specifically mentioned that they believe navigating pop-up alerts worsens their efficiency. Of those less concerned about alerts, 67% (2/3) primarily worked in the emergency department (ED) and consider frequent alerts helpful in their chaotic environment.

#### Theme 2: Automation

The second most important quality of a CDSS to end users is automation. Of the 22 clinicians, 17 (77%) believe that they should not have to search for the CDS tool; rather, it should be automatically triggered and contain prechecked orders based on EB practices. For example, medications that require renal dosing should be automatically prechecked based on the patient’s glomerular filtration rate. Instead of providing providers all possible permutations of a specific disease’s admission orders, the system should use risk stratification or other automated methods to precheck tailored orders for patients:

Boom, you can just click the box that has the dosing for the patient’s weight, everything like that, so you’re not having to go between multiple screens.

Resident MD, trauma

RNs and providers early in their careers believe that maximally automated CDSS would improve their efficiency. Of the 11 physicians, 8 (73%) concurrently pointed out that a CDSS needs to allow for clinical judgment in fitting CDS for individual patients.

#### Theme 3: Redundancy

The potential for redundancy created by CDS concerns both providers and nurses; 36% (5/14) of providers and 25% (2/8) of RNs explicitly pointed out that currently used CDS tools frequently allow for overlapping orders and therefore create confusion among nurses and added work for providers who have to clean up those orders. Diet, activity, and nursing orders were frequently cited, and medication orders were less cited:

It’s really important that this not generate a bunch of duplicate orders that have to be cleaned up, because that’s one of the number one things that is a job dissatisfier for physicians: bogus work.

MD, trauma and critical care, >10 years in practice

#### Theme 4: Minimalistic Design

Experienced providers (3/14, 21%) and some RNs (2/8, 25%) share the concern that CDS tools that are too complex (eg, poor visibility, multiple steps per task, or confusing layout) can create barriers to use. These providers value the ability to easily and quickly take in all information on a screen and have considerable disdain for EHRs that add significant complexity to their documentation experience:

I think they’re cumbersome and difficult to read. They’re hard to scroll through. Sometimes the scroll bar works, other times it doesn’t. The words are small on the screen, and you don’t see them well—they’re kind of gray instead of black—so trying to read them becomes very difficult. Then there are these paragraphs and columns, and you have multiple options, and you’re trying to read through these options for something, and you’re scrolling through, and by the time you get to the bottom, you can’t remember what the top option meant.

MD, trauma and critical care, >10 years in practice

#### Theme 5: EB

Most providers (9/14, 64%) and RNs (6/8, 75%) agreed that CDS protocols must be EB. RNs specifically desired a better understanding of the evidence behind best practices:

I think there should be on the intranet something available where everybody can scroll through to see what the research is. If somebody references it, they can quick go throw in a link to it, so we can all see what it is and it’s just there for everybody to see.

RN, critical care

Physicians believe that when CDS is supported by strong evidence, it will help standardize practice across a group, particularly when it comes to less common interventions; for example, rib fixation and nerve blocks. Other perceived benefits of linking to the evidence base include increasing buy-in from users, reducing the knowledge gap between novice and expert clinicians, and helping users of different backgrounds provide consistent care:

I think that it would serve best as an advisory tool [...] here are evidence-based recommendations that will help support this patient. It takes the guesswork, especially for moonlighting physicians or providers that aren’t really up on all the literature, and takes that information and moves it into the realm of recommendations that can standardize a practice across a trauma department.

MD, trauma, 4-6 years in practice

#### Theme 6: Promote Optimal Care and Prevent Errors

Many clinicians (8/14, 57% of providers and 5/8, 63% of RNs) believe that CDS should help them provide optimal care and avoid errors. There is an important distinction to be made with the *EB* theme, which focuses on improving adherence to the evidence and delivering evidence at the point of care. This theme focuses on how a system can prevent errors by using alerts.

In short, the EB theme guides clinicians to what they *should do*; this theme (promote optimal care and prevent errors) prevents clinicians from doing something they *should not do*:

I think something to come and say, do you really want to do that? In order for you to move forward in this, the patient has to meet these criteria and it appears that they don’t, being able to pull data from Epic to put there in front of the practitioner to say this is not in keeping with our clinical decision tool.

RN, critical care, >10 years in practice

Institutional morbidity and mortality conferences [37] and sentinel events may provide a rich resource to guide the integration of error prevention and early warning CDSSs.

#### Theme 7: Comprehensive Across a Spectrum of Disease or Injury

Half of the clinicians (6/14, 43% of providers and 5/8, 63% of RNs) share the enthusiasm for a comprehensive CDSS rather than one that addresses a single-point decision. They support comprehensive disease-specific decision support from admission to discharge. Examples include incorporating tools addressing care from admission to after discharge, predicting and addressing common complications, and facilitating a multidisciplinary approach to healing:

An ideal decision support system would be both sensitive and specific, and readily identifies those patients that you may not be thinking about, and also providing you with the options of treatment that you may not be necessarily thinking about or are knowledgeable in regards to.

MD, emergency medicine, >10 years in practice

I really think that incorporating aspects of aftercare- after hospital contact, outreach, and monitoring- would help to improve outcomes.

Resident MD, trauma

#### Theme 8: Malleability

Half of the clinicians (5/14, 36% of providers and 6/8 75% of RNs) also believe that CDSSs should be malleable and able to accommodate the needs of different facilities, phases of care, and patient populations. Providers and RNs from different specialties and hospitals expressed concerns, as well as suggestions, specific to their area of practice. This was most common among ED clinicians (5/6, 83% ED clinicians). For example, our prototype CDS included an anesthesia consult for epidural placement; however, it became apparent that this would be feasible at some hospitals but not at others:

But we don’t have a respiratory therapist, so how would that work? We don’t have a pharmacist, so how would that work for us? We don’t have a physical therapist or an occupational therapist, so I guess if the patient’s boarding in the ER, how do we make all of that happen?

RN, emergency medicine, >10 years in practice

Other areas of care that were mentioned as not falling under a one-size-fits-all model were therapies, community hospitals where providers were not in-house 24/7, and older adult populations.

Select representative quotes and CDS design recommendations are given in Multimedia Appendix 2.

**Figure 3:**
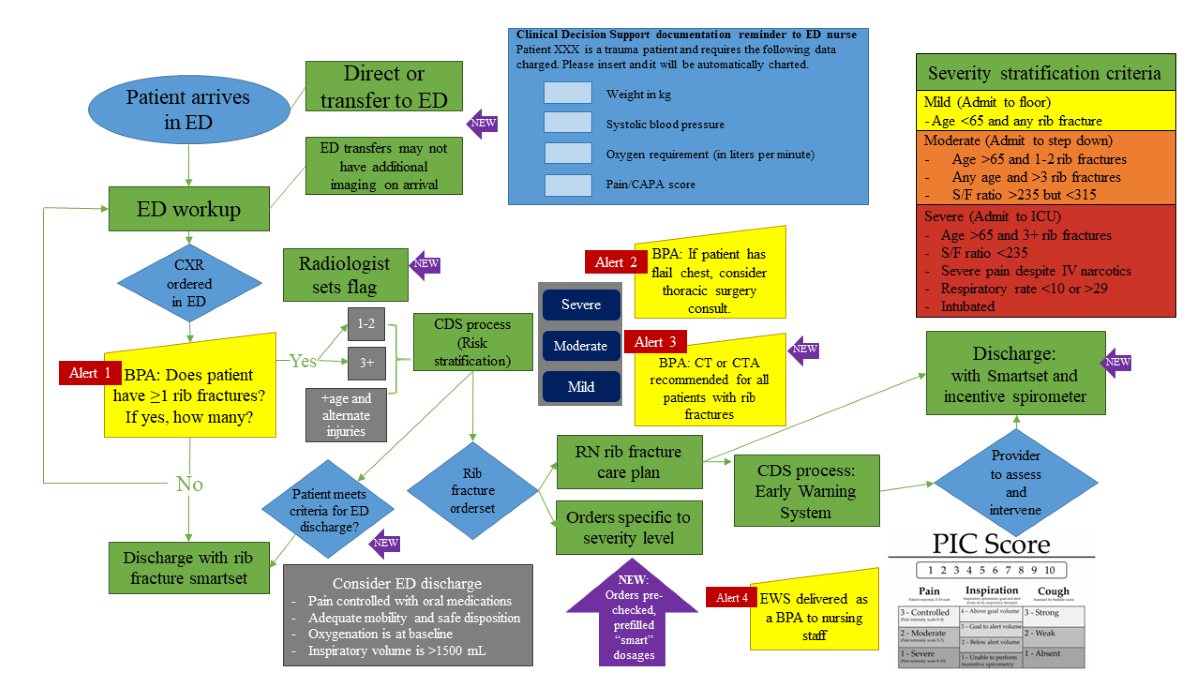
Final rib fracture clinical decision support (CDS) care map workflow (new elements designated by purple arrows). BPA: best practice advisory; CAPA: clinically aligned pain assessment; CDS: clinical decision support; CT: computed tomography; CTA: computed tomography angiography; CXR: chest x-ray; ED: emergency department; EWS: early warning system; PIC: pain inspiration cough; ICU: intensive care unit; IV: intravenous; RN: registered nurse; S/F: oxygen saturation/fraction of inspired oxygen ratio.

Using our findings from the qualitative interviews and thematic analysis, we worked with our EHR build team to implement modifications to the prototype CDS care map. For example, instead of a rib fracture multimodal analgesia panel that offers all possible analgesia options for selection *(as was initially planned in the prototype)*, end users desired a system that prepopulates analgesia based on patient risk stratification, ensures the patient does not already have duplicate medications ordered, and automatically prepopulates and prechecks the recommended nonsteroidal anti-inflammatory drug and gabapentin doses based on the patient’s renal function. Figure 3 shows the workflow for the final CDSS, with new elements designated by purple arrows.

## Discussion

### Principal Findings

**Figure 4:**
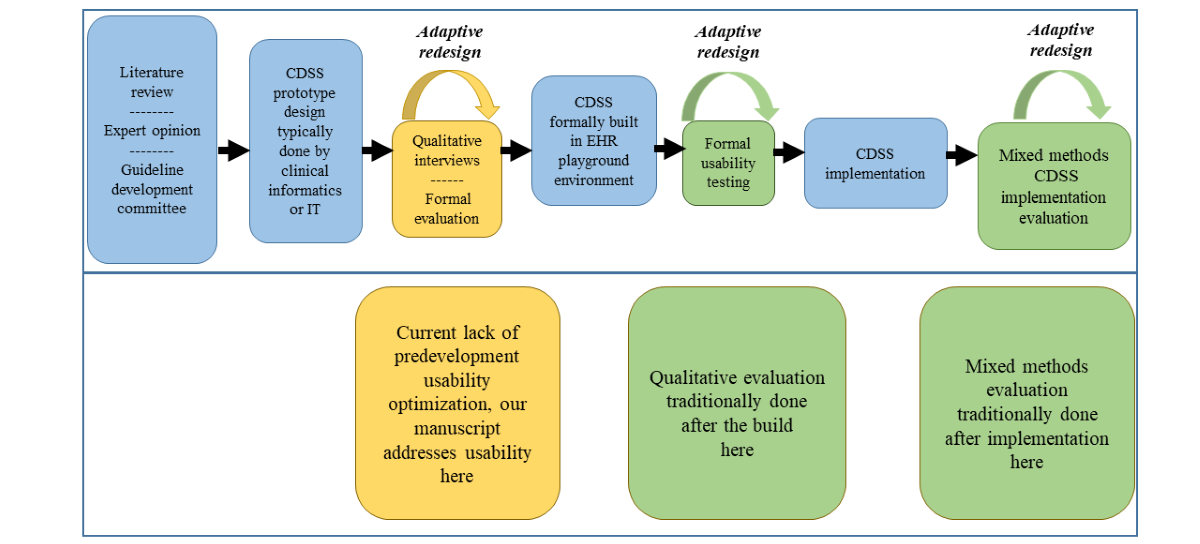
Process and sequence for the development of acceptable clinical decision support system (CDSS) care maps [2,3,13,38-41]. EHR: electronic health record; IT: information technology.

In this study, we sought to use qualitative interviews guided by the UTAUT to understand key themes associated with highly acceptable CDSSs earlier in the development and implementation process than has traditionally been done (Figure 4).

This was a planned stage of a UASAD development and implementation strategy focused on a 12-hospital system-wide CDS care map for patients with rib fractures. The following eight key themes were identified: alert fatigue, automation, redundancy, minimalistic design, EB, prevent errors, comprehensive CDS, and malleable design. Guided by these themes, an ideal CDS will provide support from ED arrival through discharge and not only focus on improving adherence with EB practice but also deliver evidence to the clinician at the point of care. Common errors or causes for patient decompensation that may occur when managing a specific disease process should be identified, and error prevention should be integrated into the design of the CDS. In addition, individuals responsible for the design and creation of such tools should take care to maximize automation and minimalistic design. CDSSs should leverage the rich structured EHR data available to provide more tailored and automated support; this facilitates minimalistic design as it reduces the necessity to deliver all possible orders to providers. Finally, as these systems are ultimately scaled across health care systems, it is imperative that they are designed to be easily tailored to individual hospital capabilities and resources while maintaining fidelity to EB practice.

To no surprise, alert fatigue was the most commonly cited barrier to CDSS acceptance. Our findings support the current literature that is increasingly recognizing alert fatigue as a negative consequence of CDSSs and a frequently cited reason for poor adherence [42,43]. A focused review of the current CDS literature has found no consensus on how to eliminate fatigue; however, previous studies agree on its contribution to poor adoption and clinician burnout [44-48]. Limiting the frequency of alerts or only assigning alerts to high-severity flags has been one solution previously proposed in the literature [49]. Our prototype CDSS included the following alerts that were active when the patient was in the ED: an alert prompting the radiologist to document whether rib fractures were present, an alert recommending a computed tomography scan for patients with a rib fracture seen on the chest x-ray, and an alert recommending surgery consultation for rib stabilization surgery in patients stratified to severe risk of complications. In addition, the admission order panel included 6 hard stops that forced provider action to get past.

We were able to make many recommendations to improve the final CDSS (Figure 3), which was based on the prototype shown in Figure 1 and redesigned based on our findings reported in this study. To combat alert fatigue, we recommended the integration of an artificial intelligence (AI)-enabled system that can *read* chest x-rays and tell the clinical team if the patient has rib fractures. AI diagnostic models using biomedical imaging are increasingly being investigated to improve diagnostic accuracy and minimize the workload of radiologists. They are used to facilitate imaging diagnosis for simple tasks and have been successfully used for several disease processes, including COVID-19 [50], acute respiratory distress syndrome [51], and pneumothorax [52] detection. We envision using a similar model for rib fracture detection. Using AI to perform the duties of identifying patients with rib fractures and quantifying the number of rib fractures present could remove these tasks from providers and decrease the frequency of alerts. To address the computed tomography scan and surgery consultation alert recommendations, we proposed a solution that monitors provider adherence with specific EB practices. In our final CDSS, clinicians that are already adherent to EB practice over a prespecified threshold will cease to receive notifications unless adherence falls below the threshold. Finally, all 6 hard stops were removed from the admission order panel. Interestingly, most of the clinicians who had expressed less concern with alert fatigue worked primarily in the ED and believed frequent alerts to be a generally positive thing and helpful in their chaotic environment. This suggests that different specialties may have different thresholds for tolerating interruptive decision support and needs further investigation.

Similarly, clinicians from all backgrounds agreed that the ideal CDSS should be maximally automated. These findings support the current literature that has shown that clinicians are hesitant to use CDSSs that require additional time and effort [53]. Another study found that automated decision support was 1 of 4 main factors contributing to the success of CDS [54]. The strongest enthusiasm for this came from younger providers who referenced their workload and need for the EHR to improve rather than hinder their efficiency. However, experienced users were more likely to mention the necessity of clinical judgment. The necessity of provider judgment in high-acuity situations has been previously identified as a challenge when designing CDS, and it is imperative to build flexibility into CDS to allow for clinician judgment [55]. Interestingly, experienced (>10 years in practice) providers were more likely to have a negative association with tools that were too complex or lengthy in appearance, suggesting that as new physicians move into practice, there will be a shift toward increased tolerance of technology complexity. In today’s EHR, when entering orders, providers frequently use *order sets* that include selectable orders related to the patient’s disease process. Historically, order sets contain lists of orders for the provider to select from; the orders are not prechecked, and thus order sets require significant time on the part of providers to decide the orders they want and then to select each individual order. By prepopulating and prechecking orders, we are providing cognitive support so providers can easily see which orders are recommended for a disease process, thereby minimizing their workload and time spent checking boxes. Although the prototype CDSS already included an automated machine risk stratification (ie, mild, moderate, and severe) that presented individualized admission order panels, in the final CDSS, we further supplemented this by automating medication dosing, order set integration, and the calculation of a rib fracture decompensation scoring system.

Historically, CDSSs have addressed individual components of a patient’s care (eg, an admission order set or ordering a specific test), which can result in disjointed care. Most CDS developed to date focus on single decision points and less on comprehensive disease-specific decision support spanning the duration of hospitalization. As a solution, we suggest creating a multifaceted CDSS that addresses care across the spectrum of a disease; this has also been suggested in the literature but has not been extensively studied [56,57]. A CDSS that incorporates all phases of care, from admission orders and imaging to discharge and follow-up, could be built to avoid redundancy as well. In addition, a CDSS with components that target different members of a multidisciplinary team may improve processes in today’s increasingly team-centered health care model [48]. To this effect, our final CDSS, which was modified based on our findings reported here, includes decision support modules for ED providers, ED RNs, respiratory therapists, the admitting team (ie, trauma surgery or internal medicine), and inpatient RNs. For ED nursing, ED decision support centers on a collection of elements critical for risk stratification, whereas inpatient nursing leverages the Epic EHR *Nurse Brain*. Decision support for inpatient providers centers on support for admission, detection of clinical worsening, and discharge; for ED providers, it assists in identifying patients with rib fractures and triaging them appropriately.

To address redundancy, our final CDSS is not delivered as another admission order set, but rather as an integrated order panel within standard admission order sets. To promote EB care, links are included in all EB guidelines or decision aids when relevant. To reduce errors and promote optimal care, medication alerts were created to trigger in response to abnormally high dosages, and medications that require renal dosing will cross-reference the patient’s glomerular filtration rate. To promote optimal care, an early warning system specifically tailored for patients with rib fractures [4,58] is integrated into the CDS to identify patients at high risk for decompensation and prompt early intervention. Finally, although the prototype CDSS was originally developed for a tertiary academic trauma center, the final CDSS was subsequently tailored to maintain fidelity but optimize logistics for various Midwest community hospitals. For example, in lieu of neuraxial blockade, regional catheters can be more easily provided at certain sites, and in lieu of the intensive care unit admission for all older adult patients with multiple rib fractures, a dedicated respiratory unit or specialized trauma floor may be used.

The integration of formal end-user qualitative feedback guided by the UTAUT model resulted in significant redesign of a trauma CDS care map and provided a framework for future prototyping. While pilot-testing or usability testing, a formal process of observing end-user interactions with a system to identify problems to repair and measure user performance [59], is an integral part of a UCD for CDS; most studies focus on UCD *after* the EHR build is complete with iterative redesign before implementation [12,18]. We believe that using qualitative assessment as a component of the UASAD model before the EHR build improves UCD by engaging end users early in the process. These recommendations are especially timely, as the realm of decision support for patients with thoracic trauma has not yet been extensively explored, with few existing CDSs reported [2,22,23].

### Conclusions

In this study, we identified the benefit of using the UASAD model in the development of an acceptable prototype CDS care map before the EHR build and formal usability testing. By optimizing user acceptance through this qualitative method of prototype design before the EHR build, the UASAD model may result in fewer iterative redesigns during usability testing and ultimately reduce development time. In addition, this approach can accommodate the input of many multidisciplinary end users, facilitating the generalizability of user acceptance. Finally, it is possible that the integration of a UTAUT-driven qualitative redesign may facilitate more substantive CDS modifications, as usability testing typically focuses on optimizing how users complete certain tasks or interact with the CDS. UASAD-adaptive CDS redesign may offer end users a blank slate to maximize acceptance and tailor initial EHR build to institutional resources and workflow. Our experience with qualitative assessment of a prototype CDS care map has helped us identify 8 themes associated with acceptable CDS that may be used as a framework for future CDS design. CDS adaptive redesign guided by end-user qualitative analysis and validated technology acceptance theories may result in systems with higher acceptability. Further research is needed to identify specific ways to incorporate these features into CDS and evaluate trends in outcomes. Our team’s next steps in the development and implementation of a rib fracture CDS care map involve performing formal usability testing on the final CDSS, iterative redesign based on findings, implementation, and assessment of outcomes.

### Limitations

Our study focused specifically on an inpatient CDS care map; therefore, these recommendations may not be generalizable for the ambulatory setting. We limited our clinicians and prototype to the trauma population; therefore, the findings may not be generalizable to nontrauma patients. Although interviewees covered both university and community hospital settings, rural and federal hospitals were not well-represented in the sample; thus, the findings may not be applicable to those settings.

## Appendix

Multimedia Appendix 1 Interview guide and script.

Multimedia Appendix 2 Representative quotes by theme.

## Abbreviations

AI: artificial intelligence
APP: advance practice provider
CAPA: clinically aligned pain assessment
CDS: clinical decision support
CDSS: clinical decision support system
EB: evidence based
ED: emergency department
EHR: electronic health record
MD: medical doctor
RN: registered nurse
UASAD: User Acceptance and System Adaptation Design
UCD: user-centered design
UTAUT: Unified Theory of Acceptance and Use of Technology
