# Supplementary material for: Barriers to and Facilitators for Acceptance of Comprehensive Clinical Decision Support System–Driven Care Maps for Patients With Thoracic Trauma: Interview Study Among Health Care Providers and Nurses
Source: JMIR Hum Factors. 2022 Mar 16;9(1):e29019. doi: 10.2196/29019 (PMC8968578; doi:10.2196/29019)
Supplement: Multimedia Appendix 1 [file humanfactors_v9i1e29019_app1.docx]

**Interview Guide and Script**

**Introduction of the interviewer**

My name is ([blinded]. I am an independent qualitative research consultant) and I am working with [blinded].

**Goals of the interview**

The goal of this interview is to better understand yours and other providers perspectives, feelings and understanding of the use of Clinical Decision Support Systems in rib fracture diagnosis and treatment.

**Disclaimer**

I am not a medical provider and I am here to learn from you. My only goal in this exercise is to learn from your expertise and accurately captures the perspectives, feelings and understanding both positive or negative.

**Consent Forms and Demographic Questionnaire**

To provide you with more detailed information about the study, its goals and procedures please take a few moments to review the following consent form. If you agree please sign off on it and complete the following brief demographic questionnaire. Your responses will be anonymous.

**Logistics of the interview**

In preparation for this interview you should have reviewed a brief 6 minute overview of the proposed Clinical Decision Support System for treating patients with rib fractures. Can you confirm if you’ve reviewed that overview? (*if yes determine when to see if info is fresh, if no, ask if they would like to take another moment to review it(bring laptop or ipad to show them?)*

My hope is to spend 45 minutes to an hour with you, and I have a series of specific questions that I would like to ask you. As we move through the questions, please let me know if a question I am asking is off, if I should be asking a different question, or if there is something you feel is more important to talk about.

There is no compensation for your time, however the results of this study will help to inform the development of future research and CDS interventions to more effectively treat patients with rib fractures. In an effort to accurately capture our conversation and information you share with me I would like to audio record our conversation.

Your identity will remain confidential/anonymous in the final analysis. The audio will only be transcribed and used in analysis however your name and any other identifying information will be removed. If you would like to share something off the record, I can pause the audio. I also plan to be taking notes by hand throughout, so as to keep track of important details that you share.

Do I have your permission to record and start the interview? (audio record this state and/or sign consent form?)

**Interview Question Guide for Rib Fracture CDS.**

*All interviewees are expected to review the CDS prior to interview.* ***A brief 6 minute review will be provided at the beginning of the interview to reinforce the proposed CDS workflow.***

| Question Type | Questions |
| --- | --- |
| Warm Up Questions | 1. Tell me about your work as a (type of medical provider) and what you most enjoy about it. 2. As you think of all the types of conditions you treat, specifically In your experience what have been some of the challenges of treating patients with rib fractures? |
| Experiences and perceptions of CDS and Application to Rib Fractures  Construct Performance Expectancy  Questions regarding perceptions of an optimal CDS in general | 1. What has been your experience using clinical decision support systems (CDS) in your practice as a healthcare provider?   Probes:   1. Are you familiar with “CDS” (Sepsis, Meds specific examples) and prevalence of use? 2. What opinions and perceptions positive, neutral and negative for you have regarding CDS and its impact on overall patient care? 3. To what extent do you believe a clinical decision support system (CDS) could be designed to support clinicians treating patients with rib fractures?   Probes:   1. Generally to what extent do you think it could impact(improve or hinder) *your efficiency/work flow for treating patients?* 2. What conceptual factors/features would be ideal and why treating rib fractures? |
| Questions regarding the proposed rib fracture CDS | **You had a chance to review the CDS model in advance of this interview and I would like to ask you some more specific questions about your thoughts about its potential impact on your overall efficiency as a provider treating patients with a rib fractures.**   1. For the proposed CDS decisions which elements would **improve or not improve your overall workflow efficiency**?   Probes:   1. Elements such as: Admission orders, admission location, (ICU/Stepdown/Floor/ED Discharge), CDS to perform rib fixation, CDS to order an epidural 2. Which of these elements would be most important/Least important?   **I’d like to now ask you some questions about your perspective of CDS and its impact on overall patient outcomes. (Walk through each applicable slide of protocol to ask specific feedback on each element)**   1. For the proposed CDS decisions previously listed, which elements could have the greatest improvement on patient outcomes?   Probes:   1. Which element, why and what specific outcomes it would improve(e.g. improved survival, reduced complications)? 2. And which elements would not improve patient outcome? 3. Are there any additional features you can think of which would further improve patient outcomes. 4. The goal would be to structure the CDS elements and pathways based on evidenced based treatment guidelines for patients with rib fractures. To what extent are you **aware of the evidence-based** recommendations / treatment guidelines for patients with rib fractures and those proposed within this CDS model?   Probes:   1. Which elements do you agree or disagree with? 2. Which elements would you want to learn more about the supporting evidence? 3. From your experience how have CDS alerts impacted your ability to provide effective patient care (positive/negative)? 4. Can you provide a specific **example(s) of good or bad alerts (communication methods) that have led to positive or negative events** *(for example failure to relay to the attending provider that a patient is decompensating, or that a patient just had a rapid response, or that a lab just came with a critical value)?* 5. To what extent do you think automated alerts during a critical event would improve your workflow? 6. All things considered when it comes to notifications, how would you envision an ideal CDS that would alert you that a patient with rib fractures requires evaluation? (i.e. pop up screen, pager, other alert?) *What types of alerts would be most useful when patients are in need of different levels of care(i.e. low to severe?)* |
| Construct Effort Expectancy: | **I’d like to now ask you some questions your perspectives on how easy it would be to use this CDS and how it should best alert you to be able to use it most effectively.**   1. How **comfortable** would you be to follow a rib fracture CDS such as the one proposed?   Probes:   1. why or why not? 2. What would make you uncomfortable and why 3. What could help to make you more comfortable (e.g. development group pilot, demonstrated lit review, testing and gaining familiarity with the interface, leadership support, etc) , 4. How **easy** do you believe it will be for you and others within your job description to use this CDS?   Probes:   1. What areas do you believe will be hard to use and which will be easy to use. 2. All things considered, aside from what we’ve already discussed, If you had to imagine the best CDS system that could support you in treating patients with rib fractures, would you add any additional features to this proposed model that might further improve your efficiency and improve patient outcomes? |
| Construct Social Influence: | **Finally I’d like to now ask you some questions about if you had access to an ideal CDS system how likely you would be to use it…**   1. To what extent do you think your boss and peers would want you to use this CDS?   Probe:   1. If yes probe which group and why? 2. If no probe which group and why 3. To what extent do you think your boss and peers would agree with the recommendations and guidelines of this CDS?   Probe:   1. If yes probe which group and why? 2. If no probe which group and why |
| Construct Facilitating Conditions: | 1. In your opinion to what extent do you feel like you **have the resources necessary to learn how to use** this CDS efficiently?   Probes:   1. If yes why, if no why not 2. What additional resources would be most useful to you to help learn how to use this CDS effectively/efficiently (e.g. time off to study it, in person individual training, group CDS training/ education) 3. If launched next week how likely would you be to use this CDS in its entirety or partially? Probe: If partially why and which elements would you not use. |
| Final Comments and Closing | **That’s all the questions that I have for now, is there anything else that you’d like to add that would be helpful as the team considers how to best design a CDS system that could be most supportive of providers in treating patients with Rib fractures?**  **Thank you very much for your time and I am going to stop the recording now. As a reminder the team may follow up directly if there are any details or questions that we might benefit from further details/ or clarifications.** |
