# Supplementary material for: Barriers to and Facilitators for Acceptance of Comprehensive Clinical Decision Support System–Driven Care Maps for Patients With Thoracic Trauma: Interview Study Among Health Care Providers and Nurses
Source: JMIR Hum Factors. 2022 Mar 16;9(1):e29019. doi: 10.2196/29019 (PMC8968578; doi:10.2196/29019)
Supplement: Multimedia Appendix 2 [file humanfactors_v9i1e29019_app2.docx]

**Multimedia Appendix 2.** Representative quotes by themes.

| **Theme and representative quotes** | **CDS^a^ design recommendations** |
| --- | --- |
| **Clinician-facing themes** | |
| **Alert fatigue** | |
| - “The other question I’ve got for you is: are you going to have a pop-up for rib fractures, another pop-up for spleen injury, another pop-up for femur fracture, another pop-up for hip fractures... where do you stop with the pop-ups? ... You might have to think about a better way to do that, because I’m not going to want to click through 25 pop-ups to write my admission orders.” [MD^b^, trauma, >10 years in practice] - “The hard-stop pop-ups are the most frustrating pop-ups because the computer does not have any judgment, so if you put a hard stop there, that computer is stopped. You can’t order any more orders on your patient until you clear the pop-up. Sometimes you end up ordering something that’s not appropriate for the patient just so you can continue with writing your orders.” [MD, trauma, >10 years in practice] - “I think BPA fatigue sets in and people just scan the screen for the dismiss button. So I think either hiding that or removing it, making sure that you have to click on something and acknowledge what the BPA is saying to you.” [APP^d^, medicine and critical care] - “I think it would depend on the problem and how severe. If it was something that needs to be addressed immediately, I would prefer if it was super-persistent, but if it was, let’s say, you need to re-check someone’s potassium after an infusion, I don’t need to know that every single second that I’m in the system, whether it’s just once a day or a banner that lives on the main screen of how we access that person’s chart. I think if it was a constant every time we logged in, it would be easy to get overlooked.” [RN^e^, critical care] | - User cannot “dismiss” an alert - Smart or customizable alerts - Time delay on alert refiring - Reserving alerts for clinicians with low adherence to EB^c^ practices - Only using “hard-stop” alerts when absolutely necessary; otherwise, leveraging “passive” alerting |
| **Automation** | |
| - “The more you can put it on auto pilot, the more it’s going to help with some of these decisions.” [MD, emergency medicine, >10 years in practice] - “Oh perfect; I’ve got everything right here. I just click this one button, I can send it out and I’ve got everything efficiently.” [RN, emergency medicine and critical care, 7-10 years in practice] - “Boom, you can just click the box that has the dosing for the patient’s weight, everything like that, so you’re not having to go between multiple screens.” [Resident MD, trauma] - “Everything is there from the anesthesia consult to imaging order, pain control. Essentially, your comprehensive needs are right there without having to flash through multiple screens, that is great.” [Resident MD, trauma] - “If things are more pre-checked, every rib fracture would come in and we’d have the ability to treat that patient to the best of our clinical decision making. So I can call RT and say I have a rib fracture, they’re going to go okay, so we can do this, this, and this with the patient before we call the doctor, get them stabilized, and now call the doctor.” [RN, >10 years in practice] | - All EB orders should be prechecked - Automatically calculate medicine dosing based on weight and renal function when relevant - Ability to document in alert itself |
| **Redundancy** | |
| - “How do you sort out this order set and a slightly competing order set for some other condition the patient has? What I see with a lot of electronic order sets is that the patient has a million orders from ten different order sets, and the nurse is stuck trying to sort out which of the competing orders in these order sets to use.” [MD, trauma and critical care, >10 years in practice] - “It’s really important that this not generate a bunch of duplicate orders that have to be cleaned up, because that’s one of the number one things that is a job dissatisfier for physicians: bogus work. [...] So, to the extent that a pre-checked order set would generate duplicate orders about things, that’s a possible problem.” [MD, trauma and critical care, >10 years in practice] - “Yeah, duplicate orders. So Epic can create limits. For example, what we have now is you’re only allowed to have one active diet order, which has its own challenges inherently. So with the new one, it will override this other one. It’s all possible, but it’s a huge build for them.” [RN, critical care, >10 years in practice] - “The other thing that helps is when I put an order in for a lab and it alerts me that there’s a duplicate, so then before I order it, I can go quick check and realize, oh, I did miss that BMP; I don’t need to order it.” [APP, medicine and critical care, 1-3 years in practice] | - The system should prevent conflicting and duplicate orders. - The system should ensure specific categories (eg, diet and activity) only have 1 order when relevant. - The system should alert if a duplicate order is placed. |
| **Minimalistic design** | |
| - “I think they’re cumbersome and difficult to read. They’re hard to scroll through. Sometimes the scroll bar works, other times it doesn’t. The words are small on the screen, and you don’t see them well—they’re kind of gray instead of black—so trying to read them becomes very difficult. Then there are these paragraphs and columns, and you have multiple options, and you’re trying to read through these options for something, and you’re scrolling through, and by the time you get to the bottom, you can’t remember what the top option meant.” [MD, trauma and critical care, >10 years in practice] - “Minimal words, not a big paragraph or something that people have to read. I have suggested that if you want information like that there could be something in there that you could click to get more information, but for the most part, I think it should be a succinct as possible.” [MD, trauma and critical care, >10 years in practice] - “To have it put into a very neat box or something that we could insert into our notes that we could just click, and that would [link] your protocol, and it would pop in all that data so we wouldn’t have to explain why we’re doing what we’re doing, because it would just be there. And mind you, it can’t be half a page’s worth of data; it needs to be five lines’ worth of data.” [APP, trauma and critical care, >10 years in practice] | - Elimination of unnecessary words and figures - Links to extra information instead of integration of that information into the best practice advisory/alert/order panel |
| **Patient-facing themes** | |
| **Evidence based** | |
| - “I think that, like I said, if you’re going to design a critical decision-making support system, and it’s going to work either as a prompt or as an informational sheet or a link or something like that, then having the evidence-based guidelines from whatever group you’re pulling from is going to help to solidify practice in an entire group.” [MD, trauma, 4-6 years in practice] - “If it gave you set data with which to approach the physician with like, ‘my BPA is telling me that their pain is not really well controlled. They’re not hitting their volumes for the incentive spirometry. They’re meeting X, Y and Z criteria, that they really should be in the ICU to receive this kind of attention so that they don’t follow this path,’ and you can support it with the evidence-based practice. I feel like that would be better utilized by the nurses.” [RN, emergency medicine and critical care, >10 years in practice] - “We’ve got a trauma dashboard. I think everybody’s got a dashboard on epic. You can put files directly into a folder there, so at 2 o’clock in the morning when you have somebody come in, you’re like, I know that we talked about rib fractures and I think this person is on the fence. What level do I consider them? They’re a highly functional 66 year old who’s still working and they don’t look like an elderly person. They don’t have a ton of comorbidities, but they’re actually moderate injury severity. Putting some of that stuff in a readily accessible spot, connected with epic would be helpful as well.” [MD, emergency medicine, >10 years in practice] - “I think there should be on the intranet something available where everybody can scroll through to see what the research is. If somebody references it, they can quick go throw in a link to it, so we can all see what it is and it’s just there for everybody to see.” [RN, critical care] | - Links to data supporting recommendation - Involvement of content experts in conception and design phases |
| **Promote optimal care and prevent errors** | |
| - “I think something to come and say, do you really want to do that? In order for you to move forward in this, the patient has to meet these criteria and it appears that they don’t, being able to pull data from Epic to put there in front of the practitioner to say this is not in keeping with our clinical decision tool.” [RN, critical care, >10 years in practice] - “Alerts to a nurse and a provider, and then with recommendations when they get the alert on how to act on it, and how soon, then to provide feedback, write a note or some kind of feedback in the alert.” [APP, trauma, 1-3 years in practice] | - The system flags inappropriate orders or doses. - Early warning systems for a worsening patient. - Warning system if there is an attempt to provide abnormal (not part of care map) care. - The warning system needs to provide recommendations; for example, if an abnormally high dose of a medication is ordered, the system should provide a recommended dose and how it arrived at that dose. |
| **Comprehensive across spectrum of disease or injury** | |
| - “An ideal decision support system would be both sensitive and specific, and readily identifies those patients that you may not be thinking about, and also providing you with the options of treatment that you may not be necessarily thinking about or are knowledgeable in regards to.” [MD, emergency medicine, >10 years in practice] - “Everyone’s on the same track, whether it be the initial provider in the emergency department, all the way up to the care teams that would take care of the patient on the floor.” [MD, emergency medicine, >10 years in practice] - “I think that for this population, if they get admitted, you better have social services and care coordinators involved from the very beginning.” [RN, >10 years in practice] - “I really think that incorporating aspects of aftercare- after hospital contact, outreach, and monitoring- would help to improve outcomes.” [Resident MD, trauma] | - CDS should be comprehensive beyond just a single discipline or single decision. - When CDS does focus on a single decision, it should consider all the disciplines that interact with that decision and provide decision support or alert multidisciplinary team members in discipline-appropriate channels. |
| **Malleability** | |
| - “But we don’t have a respiratory therapist, so how would that work? We don’t have a pharmacist, so how would that work for us? We don’t have a physical therapist or an occupational therapist, so I guess if the patient’s boarding in the ER, how do we make all of that happen?” [RN, emergency medicine, >10 years in practice] - “We have a fairly robust anesthesia department, but we simply don’t have the time to keep these people at the emergency department for somebody to get a block.” [MD, emergency medicine, >10 years in practice] - “It would need to be customizable for the particular facility or department that’s using it.” [RN, emergency medicine, >10 years in practice] | - CDS development must take into account institutional resources and workflow. - CDS must be tailored to institutional resources and workflow. - CDS must maintain fidelity to EB practice while being malleable to accommodate each institutions strengths and weaknesses. Here, it is critical to not overengineer CDS to be too malleable or too passive and lose fidelity to EB practice and decision support. |

^a^CDS: clinical decision support.

^b^MD: medical doctor.

^c^EB: evidence based.

^d^APP: advanced practice provider.

^e^RN: registered nurse.
